# Arrested development of abomasal trichostrongylid nematodes in lambs in a steppe environment (North-Eastern Algeria)

**DOI:** 10.1051/parasite/2016048

**Published:** 2016-09-09

**Authors:** Salah Meradi, Jacques Cabaret, Bourhane Bentounsi

**Affiliations:** 1 Institute of Veterinary and Agronomic Sciences, Batna University 1 05000 Batna Algeria; 2 ISP, INRA and University F. Rabelais 37380 Nouzilly France; 3 Institute of Veterinary Sciences, Constantine University 1 25000 Constantine Algeria

**Keywords:** Arrested development, Abomasum, *Teladorsagia*, Steppe Climate, Lambs, Algeria

## Abstract

Arrested development of abomasal trichostrongylid nematodes was studied in 30 permanent grazing lambs on a large farm in the North-East of Algeria. The steppe climate has cold winters and hot and dry summers. The lambs were monitored monthly for gastrointestinal nematodes using nematode faecal egg counts, from February 2008 to February 2009. Every 2 months, two of the original 30 permanent lambs were necropsied after being held in pens for three weeks so that recently ingested infective larvae could develop into adults. The highest percentage of fourth stage larvae (L4), reaching 48% of the total worm burden, was recorded in abomasal contents in June. *Teladorsagia* and other Ostertagiinae constituted the highest percentage of L4 larvae (71%), whereas the percentage of *Trichostrongylus* (17.4%) or *Haemonchus* (11.6%) remained low. The dynamics of infection observed here (highest faecal egg count in August) and the stage composition of worm burden (highest percentage of L4 in June) provide strong evidence that arrested development had occurred.

## Abstract (fr)

L’arrêt du développement chez les nématodes trichostrongylidés de la caillette a été étudié chez trente agneaux en pâturage permanents d’une grande ferme dans le Nord-Est de l’Algérie. Le climat de steppe a des hivers froids et des étés chauds et secs. Les agneaux ont été suivis mensuellement pour les nématodes gastro-intestinaux en utilisant le nombre d’œufs de nématodes fécaux, de février 2008 à février 2009. Tous des deux mois, deux des trente agneaux permanents originaux ont été autopsiés après avoir été détenus dans des enclos pendant trois semaines afin que les larves infectantes récemment ingérées puissent se développer en adultes. Le pourcentage le plus élevé de larves de quatrième stade (L4), atteignant 48 % de la charge parasitaire totale, a été enregistré dans le contenu de la caillette en juin. *Teladorsagia* et d’autres Ostertagiinae constituaient le pourcentage le plus élevé des larves L4 (71 %), alors que les pourcentages de *Trichostrongylus* (17.4 %) ou d’*Haemonchus* (11.6 %) restaient faibles. La dynamique de l’infection observée ici (excrétion fécale des œufs la plus élevée en août) et la composition en stades de la charge parasitaire (plus haut pourcentage de L4 en juin) fournit des preuves solides qu’un arrêt du développement a eu lieu.

## Introduction

The life cycle of many nematodes can be delayed by the development of free-living larval stages or an arrest of development in the host referred to as hypobiosis. This enables the parasite to have available a large number of infective forms at times in the host life cycle that coincide with the presence of susceptible young lambs, thus ensuring transmission from one generation to the next [9, 19, 23]. Lambs in the northern hemisphere are put out to graze at the beginning of spring, a time when the arrested larvae in ewes have resumed their development and have become reproductive adults shedding eggs onto pastures grazed by lambs [5, 9, 26]. Arrested development of a short or intermediate duration increases the destabilising effect on populations, whereas arrest of a duration of 5 months or more can stabilise interactions, irrespective of the regulation of the host population dynamics [16]. Arrested development in trichostrongylid nematodes is mostly recorded in regions with long cold winters [5, 9, 23, 33]. Cold winter hypobiosis is characterised by larvae in the mucosae and a long duration of more than 4 months. Dry-season hypobiosis was first described by Graber and Tager-Kagan in 1975 [20] in cattle grazed in arid zones of Niger. It was further recorded in sheep bred under tropical climates with a dry season (unfavourable for development of free-living stages) [10, 29, 38] or very dry climates such as Saudi Arabia [13]. It was later recorded in regions with dry summers and cold winters (Middle-Atlas of Morocco [7]; North-Western Syria [18]). In dry-season hypobiosis, the duration is short (1 or 2 months) and the larvae are mostly found in the abomasal content. Summer arrest of development has also been shown in various climates in the southern hemisphere, including South Africa [33] and Australia [30]. Only one type of hypobiosis has been recorded in winter in Europe [26, 34] or summer in Syria [18], or both in Morocco [7], and is related mostly to *Teladorsagia circumcincta*. The aim of this study was to evaluate the prevalence of arrested development throughout the year in a steppe environment. Due to the cold winters and dry and hot summers, we may expect that both winter- and summer-arrested development are present in this type of climate.

## Materials and methods

### Farm studied and local climate

**Figure 1.**
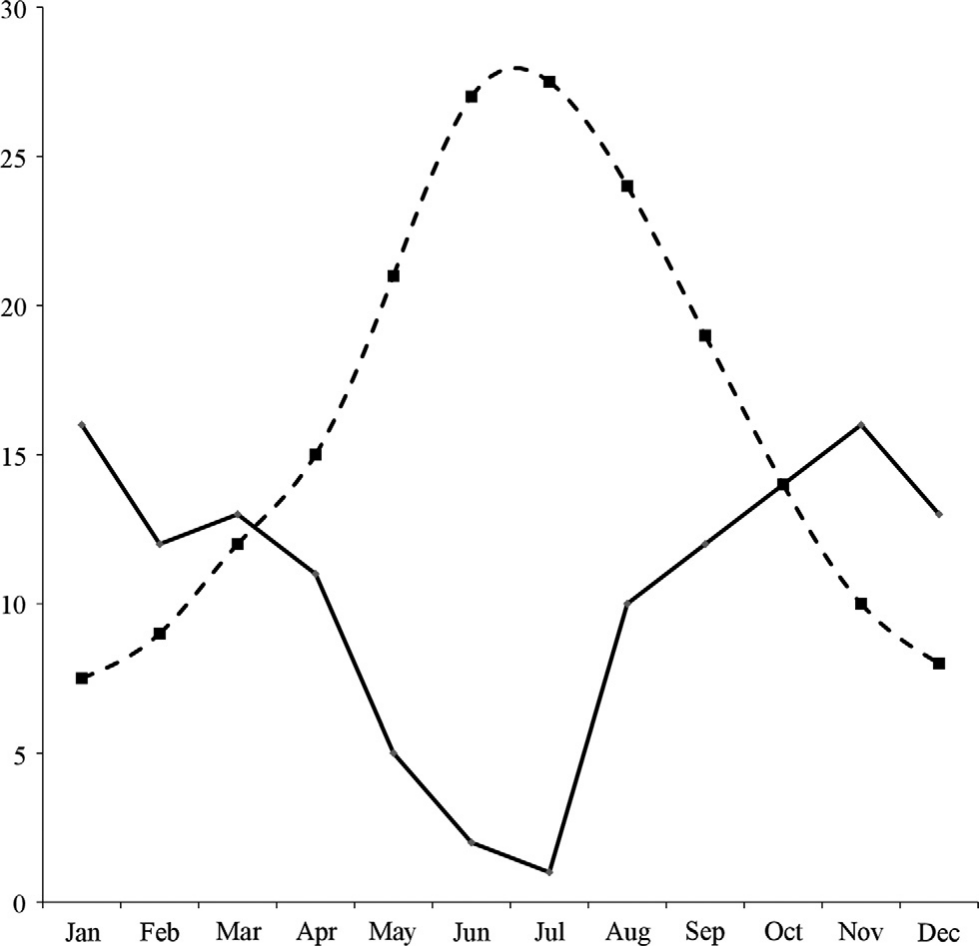
Monthly average temperature and rainfall in Batna: Data of 34 years (1974–2008) provided by the meteorological station in Batna (Algeria). Dashed line: temperature (°C); solid line: rainfall (cm).

The farm studied was located in the Batna region of North-East Algeria. The farm managed 1200 Ouled Djallel sheep, grazed partly on communal pastures with other ruminants owned by neighbouring small-holder farmers from October to May, although they were partially maintained in a sheepfold in winter. They were grazed on cereal stubble and crop residues from the end of June to September. Their grass diet was partly supplemented from October to February, and the main lambing period was in the autumn. The 1200 sheep were distributed into four independent flocks of 300 lambs. We studied one flock which was not treated during the year of study. Climatic data are presented in Figure 1. According to Emberger [14], Batna is located in a semi-arid bioclimatic zone with cold winters and could be characterised as a steppe climate according to the criterion of Viers and Vigneau [40]: steppe yearly rainfall (R in cm) is below 40 cm/year and related to yearly average temperatures (t in °C) in the following way: R < 2t. The coldest month in the area is January (average 5.3 °C) and the hottest is July (average 25 °C) based on 30-year records. The coldest months in 2009–2010 were November–February (below 5 °C for minimum temperatures), which are not favourable for the development of free-living stages [22, 28, 32]. The hottest months in 2009 were June–August (above 33 °C maximum temperatures) with rainfall below 14 cm and low relative humidity (30–40%); these conditions are not favourable for the development of free-living stages [22, 28, 32].

### Parasitological analyses

Thirty weaned lambs aged 3–4 months were selected among available lambs and were used as sentinels to monitor gastrointestinal nematode (GIN) infection between February 2008 and February 2009. They were not tracer lambs but rather permanent grazing lambs since they remained on pastures for the period; their number decreased progressively since two of them were necropsied each month. No anthelmintic treatments were given to the studied lambs during the study period. The rest of the flock also remained untreated. Faecal egg counts (number of eggs per gram of faeces: EPG) were carried out using a modified McMaster method [31] with an NaCl solution (1.18 specific gravity) sensitive to 15 EPG. When the McMaster method did not detect any eggs, a flotation method was also used, determined to be sensitive to 7.5 EPG. The eggs could be differentiated into *Marshallagia marshalli* and other gastrointestinal nematodes (GIN), the former being much larger (160–200 μm *versus* 60–110 μm, in Kassai, 1999 [21]). Every 2 months, two lambs were necropsied to identify the species of GIN and their larvae (L4 larvae and juvenile fifth stage). The lambs were held in pens for 3 weeks in order to allow the recently ingested larvae to develop into adults; the L4 larvae could then be considered as arrested since they should have developed into adults in that time. The necropsies were conducted according to MAFF (Ministry of Agriculture, Fisheries and Food) [24] and the adult GIN species were identified based on the criteria described by Skrjabin et al. [36] and morphs [8]. The larvae were identified according to Douvres (1957) [12] and Thomas and Probert (1993) [38]. The L4s were identified as Ostertagiinae since the L4 of *M. marshalli* is not described. The contents of the abomasum were sedimented in water several times until the supernatant was free of debris, and the adults and larvae of the GINs could then be identified and counted on a 1/5–1/10 aliquot. The abomasal mucosa was digested by incubation at 37 °C for 20 hr in pepsin hypochloric acid solution (10 g pepsin, 30 mL hypochloric acid and 1000 mL distilled water as described in Giangaspero et al. [18]). The resulting suspension was then sieved through a 32 μm mesh and the larvae were counted.

### Statistical analyses

The dynamics of infection intensity (based on EPG or worm counts) were analysed using the non-parametric Kruskal-Wallis test. Additionally, a logarithmic transformation for an analysis of variance (ANOVA) was carried out, followed by a *post hoc* Newman-Keuls test to classify the months into high or low infection periods based on EPG in faeces, and adults or larvae recovered at necropsy. All statistical analyses were performed using SPSS 11.5 software. The percentages of L4s and juveniles were calculated by the following formulae:

Percentage of L4s of a species=number of L4s of the species×100/total number of L4s.

Percentage of L4s in community=number of L4s×100/number (L4s+juveniles+adults).

Percentage of juveniles of a species=number of juveniles of the species×100/total number of juveniles.

Percentage of juveniles in community=number of juveniles×100/number (L4s+juveniles+adults).

## Results

### Presence of reproductive worms: dynamics of nematode egg faecal excretion

**Figure 2.**
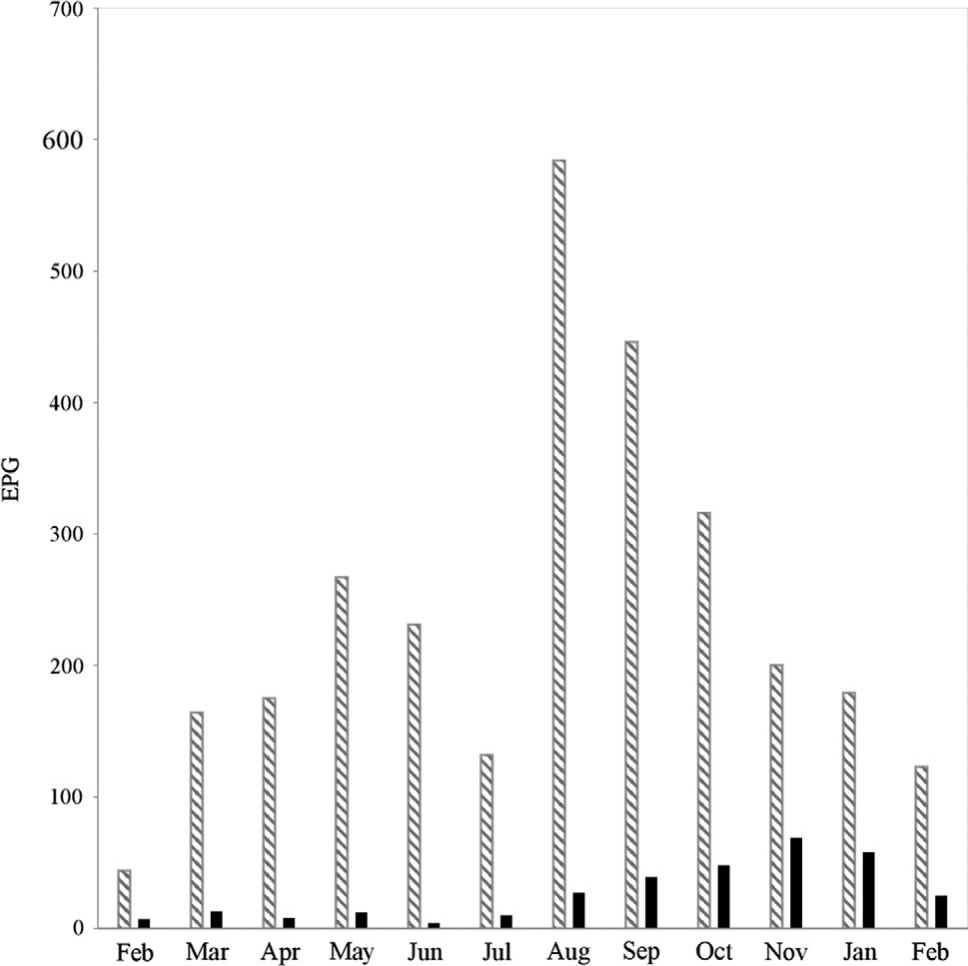
Seasonal dynamics of gastrointestinal nematode eggs per gram of faeces (EPG). Striped line: gastrointestinal nematodes; black line: *Marshallagia marshalli.*

The dynamics of faecal egg excretion of *M. marshalli* and other GINs are shown in Figure 2. According to the Kruskal-Wallis test, excretion varied significantly between the months (*p* = 0.001). The univariate ANOVA indicated that excretion of other GINs was significantly higher in August compared to all other months (*p* = 0.001). Egg excretion of *M. marshalli* was significantly higher in autumn, particularly in November (*p* = 0.02).

### Presence of several GIN species: seasonal dynamics

The different species of adult worms and their larvae observed in the six necropsies are reported in Table 1. The most abundant group of L4s observed was the Ostertagiinae in June, accounting for 71% of the L4 burden and 48% of the total worm burden (L4s/(L4s + juveniles + adults)) (Table 1). The percentage of *Haemonchus contortus* L4s in June was 11.6%. In August, the percentage of L4s in the community decreased, accounting for only 2.6% of the total worm burden, and the number of juveniles increased and reached a peak of 350 worms, representing 6.9% of the worm burden. There were two dominant adult abomasal nematode species throughout the summer period (in August): *Teladorsagia circumcincta* (*p* = 0.07) and *H. contortus* (*p* = 0.06) (non-parametric Kruskal-Wallis test). *M. marshalli* was the predominant species detected in autumn-winter. The percentage of the minor morphs of *T. circumcincta* (*T. c. trifurcata*) was 7% with variations from 4% in high-level infections (August) to 14% in low-level infections.

**Table 1.** Composition of the abomasal worm community (larvae L4, juveniles and adult worms) of lambs during the six occasions of necropsy.

Month	April	June	August	October	December	February
Mean number of adult worms						
*Teladorsagia circumcincta* morph *circumcincta*	107	670	2366	215	37	12
*Teladorsagia circumcincta* morph *trifurcata*	30	0	91	60	22	16
*Haemonchus contortus*	0	0	917	271	78	60
*Marshallagia marshalli* **	87	230	1099	1561	689	188
*Trichostrongylus vitrinus*	0	0	140	69	0	8
*Trichostrongylus axei*	0	0	0	69	6	0
Total	224	900	4613	2245	832	284
Mean number of L4s	58	860	135	0	20	0
Percentage of L4s of						
*T. circumcincta* and other Ostertagiinae	53.4	71	14.8	0	50	0
*Haemonchus contortus*	0	11.6	11.1	0	0	0
*Trichostrongylus* sp.	46.6	17.4	74	0	50	0
Percentage of L4s in community (L4s/(L4s + juveniles + adults))	20.5*	48.3	2.6	0	2.3*	0
Mean number of juveniles	0	20	350	0	10	0
Percentage of juveniles of						
*T. circumcincta* and other Ostertagiinae	0	100	100	0	0	0
*Trichostrongylus* sp.	0	0	0	0	100	0
Percentage of juveniles in community (juveniles/(juveniles + L4s + adults))	0	1.1	6.9	0	1	0

* L4s in mucosa.

** No other morph was detected.

### Arrested L4 larvae: seasonal dynamics

Statistical analyses with ANOVA showed significant differences in the numbers of Ostertagiinae and *H. contortus* fourth stage larvae (L4s) between months (*p* = 0.008; *p* = 0.001, respectively). The numbers of L4s of Ostertagiinae and *H. contortus* were found to be highest in June (ANOVA and *post hoc* Newman-Keuls test). Few L4s (L4/(L4s + juveniles + adults)) were found in lambs examined in winter (2.3% in December and 0% in February 2009). The L4s in mucosae were found in April (20.5%). They were found in the lumen at a high percentage in June (48.3%). A significant peak of juvenile Ostertagiinae was recorded in August (ANOVA, *p* = 0.01). The percentage of juveniles increased from 1.1% in June to 6.9% in August and this was an indication that L4 had resumed their development.

## Discussion

The main abomasal worm species found in the area were *T. circumcincta* and *M. marshalli*. The same fauna has also been recorded on farms in eastern Algeria by Bentounsi et al. [4], in Iraq by Altaif and Issa [1], in Syria by Giangaspero et al. [18] and in Morocco by Cabaret [7], as well as in various sites studied in a meta-analysis of the steppe region by Meradi et al. [25] or other similar regions by Suarez and Cabaret [37]. Under the same steppe climate, the faecal egg excretion dynamics found in Algeria by Bentounsi et al. [4] or Boulkaboul and Moulaye [6] showed two peaks of excretion of eggs, one in spring and one in autumn. This differed slightly from the dynamics of EPG observed in this study, as a single highest peak occurred from August to September. It is possible that these discrepancies result from the study of lambs exposed to different anthelmintic treatment regimes, as they were untreated in the present study; this was not the case in the other studies. The other reason could be that the fauna in the different sites might be different (large presence or not of intestinal *Trichostrongylus* sp., which was not evaluated in our study). The anthelmintic treatments in these areas were usually carried out in July or August when the lambs exhibit a peak of nematode egg excretion, and then in winter; all sheep are treated. The status of infection by adult GINs in European and North African studies is not very different from what has been recorded in the southern hemisphere, whether in Australia [11] or South Africa [33], where cold winters are associated with hot and dry summers. However, the presence of *M. marshalli* was not recorded. The frequency of the minor morph of *T. circumcincta* is higher than in Morocco (7% vs. 3%) but much lower than in other reports from more favourable climates where values of 20% are recorded [8].

The dynamics of the L4s (mostly *T. circumcincta*) was also seasonal, reaching a peak in June to represent 48% of the total worm burden. At this time, the L4s were found in the abomasal contents and not in the mucosa, which is the usual situation of winter-arrested larvae [3, 5, 27]. Large numbers of summer-arrested larvae have also been found in Syria [18], where they represented 85% of the total worm burden, and other regions with a long dry summer, i.e. Saudi Arabia [13], and Eastern Ethiopia [35]. In the Middle-Atlas of Morocco, both winter and summer peaks of L4s were recorded over three consecutive years [7]. The L4 summer peaks in the present study did not correspond exactly to the classification of hypobiosis in trichostrongylid nematodes given that the L4s here were not found in the mucosa [27]. However, according to Michel [26], occurrence of hypobiosis can be indicated by one of the two factors: a) the presence of the same immature stage when there has been no sudden uptake of infective larvae, and b) the continued presence of immature stages after withdrawal from infection when sheep are kept in a sheep pen longer than the pre-patent period. The results from this study clearly meet the second condition, since the lambs were kept in a pen for 3 weeks before slaughter and thus did not ingest infective larvae. It was more difficult to verify the first condition given that we had no knowledge of the availability of infective larvae on pasture. Nonetheless, the dynamics of infection observed here (highest faecal egg count in August) and the stage composition of the worm burden (highest percentage of L4 in June) provide strong evidence that arrested development occurred. This arrest appeared in June when the temperature was at its highest, the rainfall levels were negligible and the sheep were grazed on stubble fields with limited vegetation at the end of June. Both high temperatures and low moisture are extremely difficult conditions for the development of free-living stages [22, 28, 32]. It appears that larval arrest is largely triggered by climatic variables in winter [15] and probably in summer. In this study, 71% of arrested L4s were Ostertagiinae in June. It is likely that these were *T. circumcincta.* The *Marshallagia* adult population was low in summer and no hypobiosis was detected. *Trichostrongylus* sp. showed high percentages of L4s in April and August and this could also be considered as arrested development. Low levels of *H. contortus* L4s were also observed in June (11.6%), but it is unlikely that this corresponds to hypobiosis, but rather to the normal turnover of infection. This could be explained by the fact that the study region has a steppe climate and *H. contortus* is better adapted to a tropical climate [22]. Interestingly, *H. contortus* is the most commonly recorded GIN species to undergo hypobiosis during summers in dry/hot areas [2, 10, 17, 29, 39]. It is possible that the differing results in the present study may be attributed to the cold winters that interrupt the hot summers in the study area, which may explain the low percentage of *H. contortus* and the absence of summer hypobiosis for this species.

## Conflict of interest

This work was in part funded by a National Research Programme (PNR) from the Ministry of Higher Education and Scientific Research (PNR, I/U250, 233). All authors voluntarily publish this article and have no personal interest in these studies other than publishing the scientific findings that they have been involved in developing, via planning, initiating, monitoring and conducting the investigations and analysing the results.
